# SARS-CoV-2 Delta Variant N Gene Mutations Reduce Sensitivity to the TaqPath COVID-19 Multiplex Molecular Diagnostic Assay

**DOI:** 10.3390/v14061316

**Published:** 2022-06-16

**Authors:** Steven C. Holland, Ajeet Bains, LaRinda A. Holland, Matthew F. Smith, Regan A. Sullins, Nicholas J. Mellor, Alexis W. Thomas, Nathaniel Johnson, Vel Murugan, Efrem S. Lim

**Affiliations:** 1 Center for Fundamental and Applied Microbiomics, Biodesign Institute, Arizona State University, Tempe, AZ 85287, USA; schollan@asu.edu (S.C.H.); abains1@asu.edu (A.B.); larinda.holland@asu.edu (L.A.H.); msmit155@asu.edu (M.F.S.); regan.sullins@asu.edu (R.A.S.); njmellor@asu.edu (N.J.M.); 2 Center for Personalized Diagnostics, Biodesign Institute, Arizona State University, Tempe, AZ 85287, USA; alexis.w.thomas@asu.edu (A.W.T.); ntjohns2@asu.edu (N.J.); velmurugan@asu.edu (V.M.); 3 School of Life Sciences, Arizona State University, Tempe, AZ 85287, USA

**Keywords:** SARS-CoV-2, COVID-19, delta variant, N gene dropout, qRT-PCR

## Abstract

As the SARS-CoV-2 virus evolves, mutations may result in diminished sensitivity to qRT-PCR diagnostic assays. We investigated four polymorphisms circulating in the SARS-CoV-2 Delta lineage that result in *N* gene target failure (NGTF) on the TaqPath COVID-19 Combo Kit. These mutations were detected from the SARS-CoV-2 genome sequences that matched with the diagnostic assay results of saliva specimens. Full length *N* genes from the samples displaying NGTF were cloned into plasmids and assayed using three SARS-CoV-2 qRT-PCR assays. These constructs resulted in reduced sensitivity to the TaqPath COVID-19 Combo Kit compared to the controls (mean C_t_ differences of 3.06, 7.70, 12.46, and 14.12), but were detected equivalently on the TaqPath COVID-19 Fast PCR Combo 2.0 or CDC 2019_nCoV_N2 assays. This work highlights the importance of genomic sequencing to monitor circulating mutations and provide guidance in improving diagnostic assays.

## 1. Introduction

Severe acute respiratory syndrome coronavirus 2 (SARS-CoV-2) has continued to evolve through mutations in its genome throughout the coronavirus disease 2019 (COVID-19) pandemic, leading to the emergence of variants of concern (VOCs) [1,2]. SARS-CoV-2 mutations can alter antibody neutralization, change receptor binding, reduce therapeutic efficacy and reduce the sensitivity of diagnostic assays [3,4]. Although at-home, point of care, and laboratory-based antigen tests are available for rapid detection of the SARS-CoV-2 infection, qRT-PCR remains the gold standard for diagnostic testing [5].

As SARS-CoV-2 has evolved, the qRT-PCR assays used to detect it have improved. Early in the pandemic, the U.S. Centers for Disease Control and Prevention (CDC) issued a set of N gene primers to detect the presence of SARS-CoV-2 by qRT-PCR [6]. These were designed to be performed as distinct, independent reactions for each sample. Commercial SARS-CoV-2 diagnostic assays have been designed using a multiplex of multiple gene targets. The TaqPath COVID-19 Combo Kit (Applied Biosystems, Waltham, MA USA; EUA200010) targets the *ORF1ab* gene, *N* gene, and *S* gene using one primer/probe set per region. All three qRT-PCR primer/probe sets are run in a single reaction well, and the amplification of each gene is measured over an independent fluorescence channel. The more recent TaqPath COVID-19 Fast PCR Combo Kit 2.0 (Applied Biosystems, Waltham, MA, USA; EUA210384) increases gene redundancy by using primer/probe sets that target three regions in the *ORFf1a* gene, two regions in the *ORFf1b* gene, and three regions in the *N* gene. All primer/probe sets are run in a single reaction well and all targets in each genomic region (*ORF1a*, *ORF1b*, and *N* gene) share a common fluorescence channel unique to that region.

In multiplexed qRT-PCR assays, there is usually a very low difference in the threshold cycle values between the two target genes. However, mutations in the virus genome arise that can lead to poor primer or probe binding at one gene target, resulting in discordant C_t_ values between two of the gene targets, which is termed a gene target failure. Polymorphisms in the SARS-CoV-2 genome that can affect detection by commercial qRT-PCR assays have been reported for the *E* [7,8] and *N* [9] genes and other diagnostic targets [10]. For example, a 6-nucleotide deletion in the *S* gene found in SARS-CoV-2 Alpha variant leads to a failure of *S* gene target detection (*S* gene target failure, SGTF) in the ThermoFisher TaqPath COVID-19 Combo Kit real-time reverse transcription-PCR (qRT-PCR) assay. The Omicron variant also harbors the same SGTF deletion mutation, demonstrating that mutations can be recurring [11]. This failure profile has been used as a surrogate marker to estimate variant prevalence in populations [12,13].

Here, we report the characterization of mutations in the SARS-CoV-2 Delta variant genome that cause *N* gene target failure (NGTF) in the TaqPath COVID-19 Combo Kit qRT-PCR assay. This was discovered through baseline sequencing surveillance in Arizona, USA from 1 June 2021 to 21 August 2021. NGTF specimens had a greater C_t_ difference (median 7.6; IQR: 4.0) between *N* gene and *ORF1ab* gene targets, compared to non-NGTF specimens (median C_t_ difference 0.3; IQR: 1.0). Using next-generation sequencing, we identified a series of mutations associated with NGTF specimens. We performed molecular validation, demonstrating that plasmid constructs bearing these mutations led to NGTF. Finally, we show that these NGTF-associated mutations do not affect the revised TaqPath COVID-19 Fast PCR Combo 2.0 assays. Taken together, this highlights the importance of genomic surveillance and molecular diagnostics to stay ahead of the ongoing evolution of SARS-CoV-2.

## 2. Materials and Methods

### 2.1. Saliva Specimens and Diagnostic Testing

This study involved analyses of 2625 saliva specimens submitted for testing at the ASU Biodesign Clinical Testing Laboratory (ABCTL) from 1 June 2021 and 21 August 2021. Saliva samples were independently obtained by patients in 2 mL collection vials, registered, and deposited at ABCTL drop-off locations. RNA was extracted from 250 µL of saliva specimen within 33 h of sample receipt using the KingFisher Flex (Thermo Scientific, Waltham, MA, USA), following the manufacturer’s guidelines. Diagnostic testing was performed using TaqPath COVID-19 Combo Kit assay (Applied Biosystems, Waltham, MA, USA), following the manufacturer’s guidelines. Samples were determined to have an *N* gene target failure (NGTF) or *S* gene target failure (SGFTF) if the C_t_ value for that gene was 5 or more cycles higher than the *ORF1ab* gene C_t_ value. Samples were also classified as target failures if a single gene failed to amplify when a positive result was observed from the other two genes.

RNA from all the ABCTL saliva samples that tested positive for SARS-CoV-2 underwent viral genome sequencing. Sample C_t_ scores and patient metadata were obtained for the samples that generated consensus sequences. At the time of analysis, this collection included 10,134 samples, with the earliest collection date of 28 December 2020. For the sample population collected from 1 June 2021 through 21 August 2021, there were 1975 unique individuals. This study was approved by Arizona State University’s Institutional Review Board (IRB).

### 2.2. SARS-CoV-2 Genome Sequencing and Analysis

Library preparation was performed using the COVIDSeq Test (Illumina, San Diego, CA, USA) with ARTICv3 and ARTICv4 primer sets [14]. Libraries were sequenced on the Illumina NextSeq1000 (2 × 109). Sequencing reads adapter sequences were trimmed using trim-galore, aligned to the Wuhan1 reference genome (MN908947.3) using the Burrows–Wheeler aligner, BWA-MEM version 0.7.17-r1188 [15], and had their primer sequences trimmed using iVAR version 1.3.1 [16]. Pangolin version 3.1.14 [17], with its pangolin-designation libraries up to date at the time of analysis, was used to assign lineage designations. Sequence quality was validated and annotated using VADR version 1.3 [18]. Phylogenetic trees were created using the online Nexstrain analysis program (https://clades.nextstrain.org, accessed on 25 April 2022) [19].

### 2.3. Global Data Collection and Analysis

In order to evaluate global sequence prevalence, the entire GISAID.org database was accessed and downloaded through 21 December 2021. Sequences with incomplete collection dates or missing country location metadata were omitted from the analyses. The GISAID.org web interface was used to query the database for the nonsynonymous mutations found in sequence groups 641∆6 and A638G using the query strings “N_G214del, N_G215del” and “N_N213S, N_G214del, N_G215del”, respectively). To find the sequences that contain the synonymous mutations found in the sequence groups C643T and C636T, sequences were queried with “TAATGGCTGTGATGCT” and “CAATGGCTGTGATGCC”, respectively.

### 2.4. Plasmids

Plasmid constructs of the SARS-CoV-2 genomic regions were created by RT-PCR amplification on saliva RNA extracts using the SuperScript IV One-Step RT-PCR system (ThermoFisher, San Diego, CA, USA) and primers derived from the ARTIC V4 primer set (SARS-CoV-2_95_LEFT: GTGCGTTGTTCGTTCTATGAAGAC; SARS-CoV-2_98_RIGHT: TTTAGGCCTGAGTTGAGTCAGC). DNA bands were gel excised and used as a template for dA-tailing using DreamTAQ (ThermoFisher, San Diego, CA, USA) polymerase. The fragments were then ligated into pCRII-TOPO (ThermoFisher, San Diego, CA, USA) vectors. All cloned constructs were verified by Sanger sequencing.

### 2.5. Real-Time Quantitative Reverse Transcription PCR (qRT-PCR) Assays

The qRT-PCR assays TaqPath COVID-19 Combo Kit, TaqPath COVID-19 Fast PCR Combo 2.0 and CDC 2019_nCoV_N2 primer/probe sets were performed. For the CDC 2019_nCoV_N2 assay, each reaction contained the following: 4.5 μL H_2_O, 12.5 μL SuperScript III One-Step mastermix, 1.0 μL forward primer, 1.0 μL reverse primer, 0.5 μL CDC 2019_nCoV_N2 probe, 0.5 μL SuperScript III Taq, 5 μL sample. For the TaqPath COVID-19 Combo Kit assay, each reaction contained the following: 10 μL H_2_O, 6.25 μL TaqPath 4x MasterMix, 2.5 μL 1:10 dilution MS2, 1.25 μL COVID-19 assay, 5 μL sample. For the TaqPath COVID-19 Fast PCR Combo 2.0 assay, each reaction contained the following: 9 μL H_2_O, 5 μL MasterMix, 1 μL FastPCR, 5 μL sample. All assays were performed on a QuantStudio 7 flex (Applied Biosystems, Waltham, MA, USA) and analyzed with QuantStudio Real-Time PCR Software v1.7.1 (Applied Biosystems, Waltham, MA, USA). Run methods provided by the manufacturer were used in the TaqPath assays and for the CDC 2019_nCoV_N2 assay, the following method was used: hold stage, 1 cycle involved 25 °C, 2 min; 53 °C, 10 min; 95 °C, 2 min. The PCR stage, 40 cycles involved 95 °C, 3 s; 60 °C, 30 s. The CDC 2019_nCoV_N2 probe was detected by FAM dye fluorescence and C_t_ values were determined using a fluorescence threshold of 140,000. The TaqPath COVID-19 Combo Kit and TaqPath COVID-19 Fast PCR Combo 2.0 *N* gene probes were detected by VIC dye fluorescence and C_t_ values were determined using a fluorescence threshold of 200,000.

## 3. Results

### 3.1. Identification of SARS-CoV-2 N Gene Target Failures

**Figure 1:**
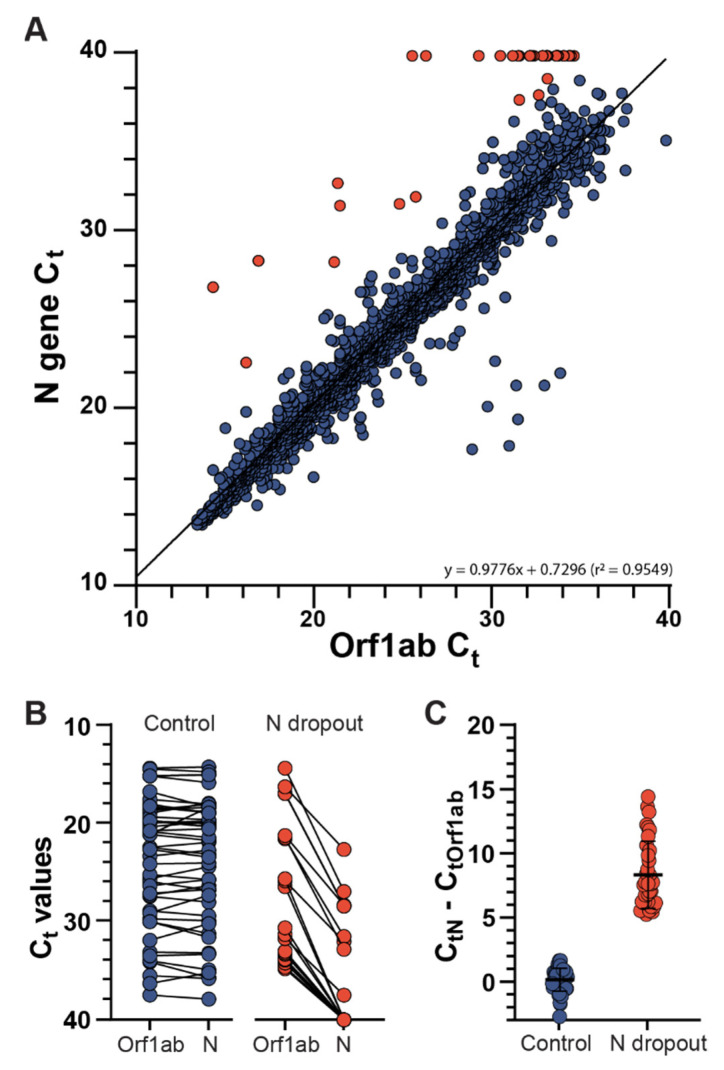
(**A**) *ORF1ab* and *N* gene TaqPath qRT-PCR Ct values for saliva samples submitted to ABCTL during the time period of 1 June 2021 through 21 August 2021 (*n* = 2625). Samples not meeting target failure criteria are colored blue, samples displaying *N* gene dropouts are colored red. (**B**) *ORF1ab* and *N* gene C_t_ values of the control group, comprised of 50 random members sub-sampled from the complete data set, are shown in blue. C_t_ values of the *N* gene dropouts are shown in red. (**C**) Difference in *N* gene and *ORF1ab* genes of the control and *N* dropout groups.

We analyzed 2625 SARS-CoV-2-positive saliva specimens from Arizona, USA (collection dates between 1 June 2021 and 21 August 2021) that were tested with the TaqPath COVID-19 Combo Kit assay. This represents Arizona populations from Maricopa (63.89%), Coconino (20.38%), Pima (9.30%), Pinal (1.71%), Yavapai (0.49%), Navajo (0.11%), Yuma (0.11%), Mohave (0.04%) and San Juan (0.04%) counties. Most specimens had concordance between the *N* gene and *ORF1ab* gene target C_t_ values (Figure 1A, blue circles, r^2^ = 0.9549). However, 39 specimens (1.49%) had *N* gene target failure (NGTF). NGTF was defined as qRT-PCR amplification failure of the *N* gene target (indicated as a C_t_ value of 40), or an *N* gene C_t_ value that deviated five or more cycles from the *ORF1ab* gene (Figure 1A, red circles). The NGTF specimens had a greater C_t_ difference (median 7.6; Q1: 6.20, Q3: 10.20) between the *N* gene and other gene targets compared to a random subsampling of non-NGTF specimens (median Ct difference 0.3; Q1: −0.43, Q3: 0.63) (Figure 1B,C, *p* < 0.001). *S* gene target failure (SGTF) was observed in 144 specimens (5.49%) consistent with the Alpha variant (B.1.1.7 lineage) circulating in this time period, and 8 specimens (0.3%) had *ORF1ab* gene target failure.

### 3.2. Next-Generation Sequencing Analysis of Delta Variant N Gene Dropouts

**Figure 2:**
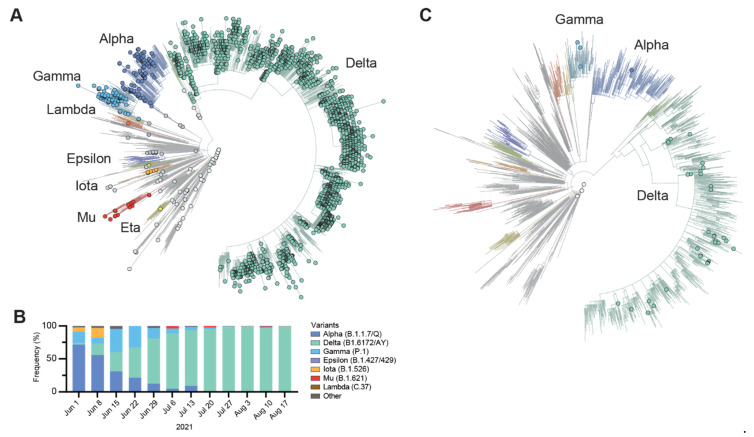
(**A**) Phylogenetic tree of 2332 SARS-CoV-2 positive samples sequenced by ABCTL (spheres) and 2493 reference genomes (unmarked). Samples are colored by their lineage designation. Tree visualization and reference genome curation was performed using Nextstrain [19]. (**B**) SARS-CoV-2 lineage frequency for Arizona of the study time period. (**C**) Phylogenetic tree of the 37 *N* gene dropouts (spheres) and 2493 reference genomes (unmarked).

To understand the evolution of circulating SARS-CoV-2 variants, we performed baseline surveillance genomic sequencing. A total of 2332 SARS-CoV-2 genomes were successfully sequenced, the majority of which were the Delta variant (B.1.617.2 and AY sub-lineages) (90.8%) (Figure 2A). Other variants within this study period included the Alpha variant (B.1.1.7) (4.6%), Gamma variant (3.3%), Mu variant (0.6%), Iota variant (0.3%), and other variants that each comprised less than 0.1% of the total samples (Figure 2B).

**Figure 3:**
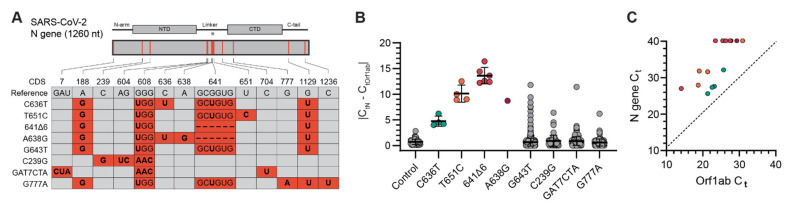
(**A**) Diagram of the *N* gene polymorphisms found in the 8 sequence groups and their location within the *N* gene. A red box indicates the presence of that polymorphism in the sequence group, a gray box indicates its absence. The asterisk (*) above the *N* gene cartoon indicates the area of interest around G643 investigated using synthetic constructs (see Figure 4). (**B**) Absolute difference in *N* gene and *ORF1ab* C_t_ values for each sample group and the control group. (**C**) *ORF1ab* C_t_ values vs. *N* gene C_t_ values for sequence groups exhibiting NGTF behavior.

Within this dataset, we obtained whole genome sequences for 37 NGTF specimens (33 Delta variants, 3 Gamma variants, 1 Alpha variant, 3 non-VOC lineages). We identified eight N gene mutation profiles associated with NGTF specimens (Figure 3A). We queried our collection of 10,134 SARS-CoV-2 genomes using these mutation profiles and compared qRT-PCR C_t_ values associated with these mutation profiles. Four NGTF sequence groups had C_t_ values that significantly deviated from the following controls: C636T, T651C, and 641∆6, and A638G (Figure 3B; *p* < 0.001; Mann–Whitney). These samples had *ORF1ab* C_t_ values of <32, indicating that low viral load was unlikely to be a factor in NGTF (Figure 3C). The other four additional *N* gene sequence groups (G643T, C239G, GAT7CTA, G777A) did not significantly vary from the control C_t_ values (Figure 3B, gray).

### 3.3. Molecular Validation of NGTF Mutations

**Figure 4:**
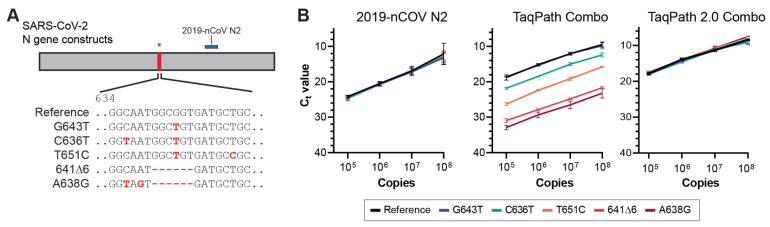
(**A**) Map and nucleotide sequence of constructs used to validate sequence dependent *N* gene target failures. The listed sequences begin at nucleotide 28,907 (*N* gene nucleotide 634) as mapped to the Wuhan reference strain. Bases colored red are mutations from the Wuhan reference strain. The CDC 2019-nCoV_N2 amplicon region is denoted with a blue box. The asterisk (*) above the N gene cartoon indicates the area of interest around G643 investigated using synthetic constructs. (**B**) qRT-PCR threshold cycle values performed on plasmids containing cloned *N* genes. Samples were assayed using the CDC-recommended 2019_nCoV_N2 primer/probe set (left), TaqPath COVID-19 Combo Kit (middle), or TaqPath COVID-19 Fast PCR Combo Kit 2.0 (right).

To molecularly validate the NGTF mutations, we cloned the *N* gene from the specimens that harbored the C636T, T651C, 641∆6, and A638G mutations (Figure 4A). As controls, we included plasmid constructs containing the Wuhan1 reference sequence and the Delta variant sequence G643T. First, we performed the 2019_nCoV_N2 qRT-PCR assays that targets a region of conserved sequences in the plasmid panel (nucleotides 891–957). As expected, serial dilutions of plasmid constructs in the panel performed similarly in the 2019_nCoV_N2 assay (Figure 4B, left). We next performed the TaqPath COVID-19 Combo Kit assay. Both the reference and G643T constructs were equally detected. However, the NGTF mutation constructs had marked shifts in C_t_ values (Figure 4B, center). Construct A638G was the most impacted (mean difference 14.12 ± 0.87), followed by construct 641∆6 (mean difference 12.46 ± 0.41), construct T651C (mean difference 7.70 ± 0.54), and construct C636T was the least impacted (mean difference 3.06 ± 0.33). Finally, we tested the TaqPath COVID-19 Fast PCR Combo Kit 2.0 that was redesigned with multiple target redundancy per gene. All plasmid constructs, including the previously associated NGTF mutations, performed equivalently without a significant shift in C_t_ values (Figure 4B, right). These results indicate that *N* gene mutations between nucleotides 636 and 651 affect the TaqPath COVID-19 Combo Kit. However, they did not impact TaqPath COVID-19 Fast PCR Combo Kit 2.0 performance.

### 3.4. Global Incidence of Delta Variant N Gene Dropout Mutations

**Figure 5:**
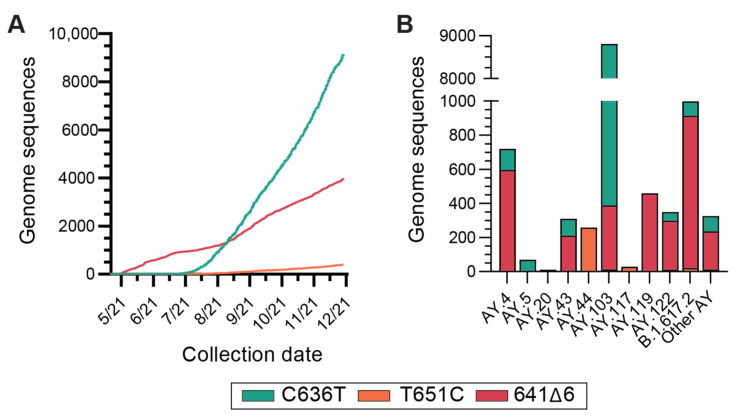
(**A**) Cumulative number of samples contained in the GISAID database that contains the NGTF mutations belonging to the Delta lineage. (**B**) PANGO sub-lineage designations of samples contained in the GISAID database containing the NGTF mutations. The parent Delta classification (B.1.617.2) and top five AY sub-lineages for each mutation have been plotted.

We next investigated the global prevalence of these NGTF mutations by querying the sequences deposited in the GISAID database through 1 December 2021 (6,297,034 samples). There were 3,619,255 Delta lineage samples. The B.1.617.2 parent lineage contained 171,882 samples and 3,447,373 samples belonged to Delta AY sub-lineages. The A638G motif was found in four (<0.01%) samples, all of which were Delta sub-lineages. The 641∆6 motif was found in 3986 (0.11%) Delta sub-lineage genomes (Figure 5A) and 241 (<0.01%) non-Delta sub-lineage genomes. It was found predominantly in the B.1.617.2 sub-lineage (Figure 5B). The C636T motif was found in 9194 (0.25%) Delta sub-lineage genomes (Figure 5A). It was found primarily in the AY.103 sub-lineage (Figure 5B). The T651C motif was found in 401 (0.011%) Delta sub-lineage genomes (Figure 5A). It was found primarily in the Delta AY.44 sub-lineage (Figure 5B).

## 4. Discussion

In this report, we identified the SARS-CoV-2 Delta lineage mutations responsible for NGTF on the TaqPath COVID-19 Combo Kit. We performed molecular validation experiments to demonstrate that they caused NGTF on the TaqPath COVID-19 Combo Kit. We also showed that the updated TaqPath COVID-19 Fast PCR Combo 2.0 assay was not affected by these mutations. These results demonstrate the importance of genome sequencing in diagnostics to account for SARS-CoV-2 evolution.

The functional consequences of the NGTF mutations are not well understood. The NGTF mutations are located within the highly disordered linker region within the N protein that is adjacent to the serine-arginine (SR) rich motif [20]. Both the 641∆6 and A638G mutations result in a two amino acid deletion of G214 and G215 at the edge of a predicted B cell epitope [21]. Mutations at position G215 have been previously implicated in viral transmissibility [22], whereas C636T and T651C are synonymous mutations.

Studies investigating target failure have been reported for the *E* [7,8] and *N* [9] genes, as well as other diagnostic targets [10]. The 641∆6 mutation has been shown to cause NGTF in the nucleic acid amplification-based Allplex SARS-CoV-2 Assay, but N protein was still detected using the FREND COVID-19 Ag rapid antigen test [23]. This study expands upon these previous studies by including molecular validation, in addition to bioinformatic analysis.

Due to the proprietary nature of the TaqPath assays, we are limited in our ability to confirm where the NGTF mutations lie in relation to the assay primers and probe. Nonetheless, our molecular validation experiments demonstrate that they interfere with the TaqPath COVID-19 Combo Kit assay performance. Constructs with the *N* gene polymorphisms performed equivalently to the controls on the TaqPath COVID-19 Fast PCR Combo 2.0 Kit assay. The uncertainty in the primer binding location limits confirming whether this is because fluorescence from the other two *N* gene targets compensates for the target failure at a single locus, or because the assay no longer targets the nucleotide region used in the original assay.

This study highlights the public health benefit of genomic sequencing in epidemiological surveillance. Pairing diagnostic C_t_ values with genome sequences provides the ability to identify polymorphisms that may evade diagnostic assays. As SARS-CoV-2 continues to evolve, new mutations and variants are expected to arise. Knowledge of their effect on diagnostic assays is important, as novel variants of concern may arise with recurrent mutations [11].

## Data Availability

SARS-CoV-2 genome sequences have been made publicly available in the GISAID global EpiCoV database (http://www.gisaid.org; accessed 20 December 2021).
